# Acute effects of footwear and surface condition on sport specific performance in athletes

**DOI:** 10.1038/s41598-025-91515-w

**Published:** 2025-02-26

**Authors:** Stanislav Dimitri Siegel, Mareike Sproll, Astrid Zech

**Affiliations:** Department of Human Movement Science and Exercise Physiology, Institute of Sport Science, Friedrich Schiller University Jena, Seidelstraße 20, 07749 Jena, Germany

**Keywords:** Minimalist shoes, Barefoot, Shoes, Footwear, Performance, Sex, Musculoskeletal system, Environmental impact, Biomedical engineering

## Abstract

This study examined the acute effects of minimalist shoes, standard sport shoes, and barefoot conditions on sports-specific performance in forty-eight team and racket sport athletes across three testing sessions. Biomechanical laboratory assessments included 90° cutting maneuvers (90°COD) and jump tests. Linear (LS) and multidirectional sprint (MS) performances were evaluated in a second session on an indoor sports floor. In the third session, LS and MS performance were tested exclusively in minimalist shoes on tartan and artificial turf. Performance was quantified as test completion time and jump height. Significant footwear effects were observed for 90°COD, MS, and LS, with minimalist shoes and barefoot conditions reducing performance by up to 9% compared to standard sport shoes. Jumping performance was unaffected by footwear. Surface conditions significantly influenced LS and MS, with tartan and artificial turf improving performance by up to 9% compared to indoor surfaces. Significant sex differences were observed in all tests except for 90°COD. These findings suggest that the effects of footwear are context-dependent and should not be generalized without considering specific surfaces, movements, and individual factors. Future research should investigate whether long-term habituation can alter these outcomes.

**Supplementary Information:**

The online version contains supplementary material available at 10.1038/s41598-025-91515-w.

## Introduction

Barefoot gait has gained popularity among the general population in recent years, largely due to the growing body of research and preliminary evidence of its potential health benefits. Meta-analyses have shown that barefoot gait leads to favorable changes in biomechanical variables^1,2^, foot morphology^3^ and improvement in running economy^4^. However, walking or running barefoot is not always feasible due to challenging ground and weather conditions. As a solution, minimalist footwear has been developed to simulate barefoot conditions. These shoes are defined as “footwear providing minimal interference with the natural movement of the foot due to its high flexibility, low heel to toe drop, weight and stack height, and the absence of motion control and stability device”^5^. Minimalist footwear is expected to promote biomechanical and morphological adaptations comparable to those observed for barefoot conditions^6–8^.

Although the impact of minimalist footwear on walking and running biomechanics is well investigated, its effects on specific athletic movements such as jumping, sprinting, and cutting maneuvers remain less understood. The interaction between the athlete’s foot, footwear, and the surface is crucial for the execution of these movements, as this is the point where the force generated by the muscles is transferred to the ground^9,10^. From a biomechanical standpoint, the direction and magnitude of the braking and propulsion forces applied during the ground contact are essential for performance^11–14^. It has been shown that specific shoe properties such as shoe mass^15,16^, forefoot bending stiffness^17,18^, outsole traction^14,16,18^, and upper configuration^19,20^ can create favorable biomechanics for achieving optimal performance in a variety of athletic movements. However, minimalist footwear is characterized by unique properties that differentiate it from traditional sports footwear. While traditional athletic shoes are designed to provide cushioning, stability, and motion control^10^, minimalist shoes promote a more natural foot motion by reducing structural support and heel-to-toe drop. This design may enhance proprioception but could also influence movement efficiency and stability in high-impact or multi-directional athletic tasks^21^. While minimalist shoes may not necessarily contain technical elements as traditional sports shoes, there is a lack of clarity regarding their ability to support athletic movements optimally^22^.

Studies that investigated the acute effect of minimalist shoes compared to standard sport shoes on jump performance have not yielded consensus^23–26^. While LaPorta, Brown^27^ reported an improvement in jumping height with minimalist shoes, Sinclair, Toth^28^ noted a decline. Possible reasons for the controverse findings in previous studies could be the rather small sample size, the heterogeneity of participants, and the broad range of footwear that not always met the standard definition criteria of minimalist shoes^25,27,29^. Moreover, the few studies on the acute effects of various types of footwear during linear sprinting^14,18,30–32^ and change of direction movements^17,18,29,33–36^ primarily focused on biomechanics but did not report effects for athletic performance. Consequently, there is still considerable uncertainty as to whether the minimalist footwear or barefoot condition might influence or even impair performance during linear and non-linear athletic movements.

The primary objective of this study is to investigate the acute effects of minimalist vs. standard and no footwear on jumping, sprinting, and change of direction performance in male and female athletes. In addition, the potential interaction between minimalist footwear and different surface conditions was analyzed, providing further insight into the feasibility of minimalist footwear for team and individual sports. The study addresses several key hypotheses:

1. Firstly, the performance measures are expected to be influenced by the type of footwear.
2. Minimalist footwear is anticipated to affect athletic movements differently. While it may be beneficial or without negative effects for more linear movements like sprinting and jumping^15,16,37,38^, it could have detrimental effects in activities that require greater stability^21,39^, such as changing direction.
3. Surface conditions are hypothesized to significantly influence performance when using minimalist footwear, potentially amplifying or mitigating its effects.
4. Lastly, gender differences in response to minimalist footwear are expected. These differences may be attributed to sex-specific variations in biomechanics^40–42^, and anthropometrics, like body mass^43,44^, foot morphology^45,46^, and muscle strength^47,48^.

## Methods

A randomized crossover study was conducted in which all participants underwent three test sessions in a consistent order over the 6-month study period. Participants were instructed to arrive at the laboratory at least 3 h postprandial, fully hydrated and to avoid strenuous exercise in the 48 h prior to a testing session. All measurements took place on University facilities. The initial session took place in the biomechanics laboratory. The second session was held in an indoor sports center on indoor sports surface. The third session was outdoor on a tartan track and artificial turf. Three footwear conditions were compared: standard sport vs. minimalist vs. barefoot. The sequence of the footwear and surface conditions was randomized, while the same test order was consistently maintained for each participant. All measurements as well as the placement of the measuring instruments were executed by the same scientists.

Active, healthy, habitual shod females and males were recruited from local sports clubs and the university setting through a flyer and word-of-mouth. All participants were required to be actively competing in a non-barefoot sport at the time of the study. Participants were excluded from the study if they had a lower extremity injury in the last 6 months, a clinically confirmed foot deformity, motor-functional impairment, or if the participant was already wearing minimalist footwear regularly prior to the start of the study. Ethical approval was obtained from the local university ethics committee (protocol number FSV 22/066). Informed consent was obtained from all subjects and/or their legal guardian(s). All participants were fully informed about the study and voluntarily agreed to participate by signing a written consent form. The study adhered to the principles outlined in the Helsinki Declaration to ensure ethical conduct throughout the research process.

### Footwear and surface conditions

**Fig. 1:**
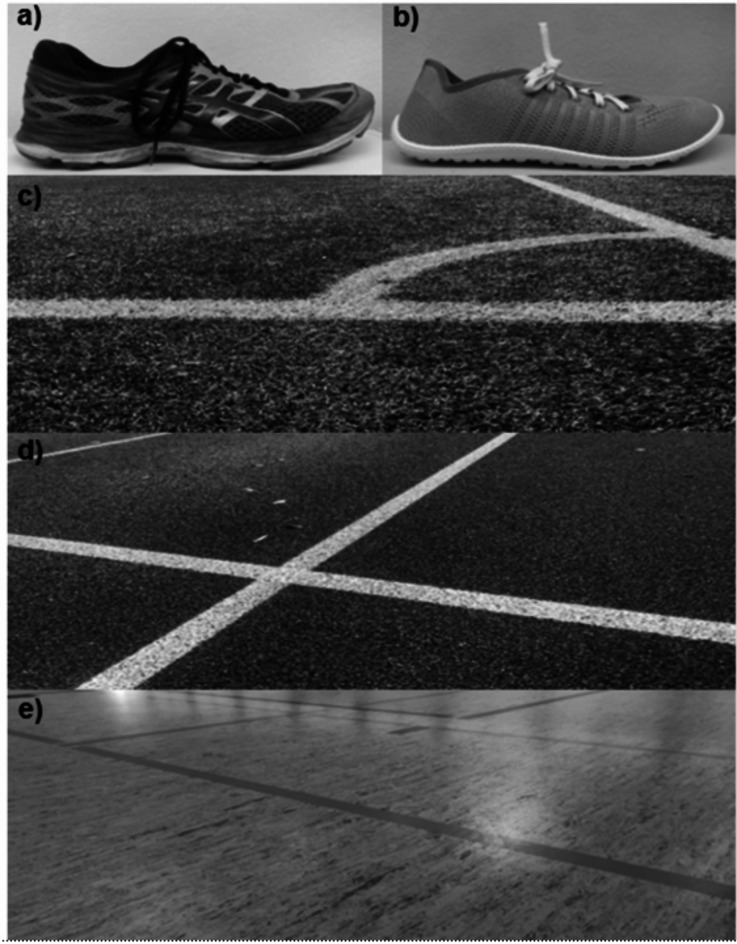
(**a**) Example of a standard sport shoe (**b**) leguano GO (**c**) artificial turf (**d**) tartan surface (**e**) indoor surface.

The footwear conditions were categorized into barefoot, minimalist shoe (leguano GO, leguano GmbH, Germany), and standard sport shoe (Fig. 1a,b). For the standard sport shoe condition, participants were instructed to use their own habitual cushioned sport shoe. The standard sport shoes were photographed prior to the measurement and were subsequently categorized based on their functionality (Appendix, Table 1). The surface conditions included indoor flooring, tartan, and artificial turf (Fig. 1c–e), as these are commonly used in sports and provide a realistic representation of typical playing environments. The tartan surface (Spurtan WS, Polytan GmbH, Germany) is a water-permeable surface certified by the International Association of Athletics Federations (IAAF) with a coating of rubber granules and elastomer. A FIFA (Fédération Internationale de Football Association) laboratory-tested surface from Tarkett Sports (Paris La Defense Cedex, France) was used for the artificial turf. This turf integrates a thermal-bonded cross-linked polyethylene foam layer (Proplay 20, Schmitz Foam Products B.V., Netherlands), silica sand as stabilizing infill, styrene-butadiene rubber as performance infill, and is tufted. The indoor floor (Sportbodenbau Kupries GmbH, Rattelsdorf, Germany) consists of linoleum and polyurethane (PUR), which meets the requirements for sliding friction according to DIN 18,032.

**Table 1:** Sample mean characteristics for all participants.

	Females *n* = 17	Males *n* = 31	All *n* = 48
Age (year)	20.71 ± 2.49	22.16 ± 4.71	21.65 ± 4.10
Height (cm)	167.91 ± 4.52	182.00 ± 7.73	177.01 ± 9.57
Body weight (kg)	62.71 ± 8.41	79.63 ± 11.74	73.64 ± 13.38
BMI (kg m^− 2^)	22.21 ± 2.54	23.97 ± 2.76	23.34 ± 2.79
Years of sports activity (year)	9.97 ± 5.00	14.26 ± 5.13	12.68 ± 5.49

*BMI* body mass index.

### Procedure

The first test session in the laboratory included three tasks with each footwear condition: countermovement jump (CMJ), single leg drop jump (DJ), 90° change of direction (90°COD). All tests were performed on two embedded force plates (1.70 × 0.5 m) (Bertec Corporation, Ohio, USA) and sampled at 2700 Hz. Jump height as well as the execution times of the change of direction tasks were used for analysis. The warm-up and familiarization phase were standardized and consisted of a total of 20 trials of the 90°COD with a progressive increase in intensity, followed by familiarization in the CMJ and DJ exercises. The second test session in the indoor sports center included two tasks: a 25 m multidirectional sprint (MS) and a 30 m linear sprint (LS) using all footwear conditions. The third test session was performed outdoors on a tartan track and adjacent artificial turf only in minimalist shoes. Both tasks in both sessions were preceded by 10 multidirectional sprints, with a progressive increase in intensity as part of the warm-up and familiarization phase. Additionally, a familiarization with linear sprinting was conducted. During both sessions, a wireless infrared timing system (TC, Brower Timing Systems, Draper, UT, USA) consisting of three photoelectric sensors, TCi Motion Start and TCi Timer, was used. The photoelectric sensors were installed on tripods, elevated 110 cm from the ground. Running times were captured at 10 m, 20 m, and 30 m times.

All tests were explained and demonstrated by the same examiner. Feedback was given if the test was performed invalidly. A break of at least one minute was ensured between each trial and five minutes between footwear/surface conditions. The sequence of performance tests and the starting leg were randomized to prevent systematic fatigue accumulation and potential learning effects. This randomization approach helps control for order effects, ensuring that performance differences are not biased by test sequence.

#### Bilateral jump performance

To evaluate bilateral jump performance, a CMJ was conducted. Each foot was placed on one force plate, the hands were on the hips. Participants were instructed to jump as high as possible and to land as closely as possible to the point of take-off. Each participant completed three valid trials.

#### Single-leg drop jump performance (DJ)

Participants completed three valid trials with each leg of the DJ according to O’Connor^49^. In DJ, participants stood in a single-leg stance on a 20 cm box, then jumped from a distance 25% of their height and landed on a force plate with the same leg. This distance was chosen to standardize the horizontal component of the drop jump across participants based on their individual anthropometrics, as recommended by O’Connor^50^, to ensure biomechanical comparability and consistency. Immediately after landing, the participant performed a single-leg maximum vertical jump with freely moving arms. A trial was considered invalid if the participant did not jump off with one foot, did not jump off the box vertically, did not land with the whole foot on the force plate, touched the ground with the swing leg, lost/fell off balance, or did not complete the task in a fluid motion.

#### 90° cutting maneuver performance (90°COD)

**Fig. 2:**
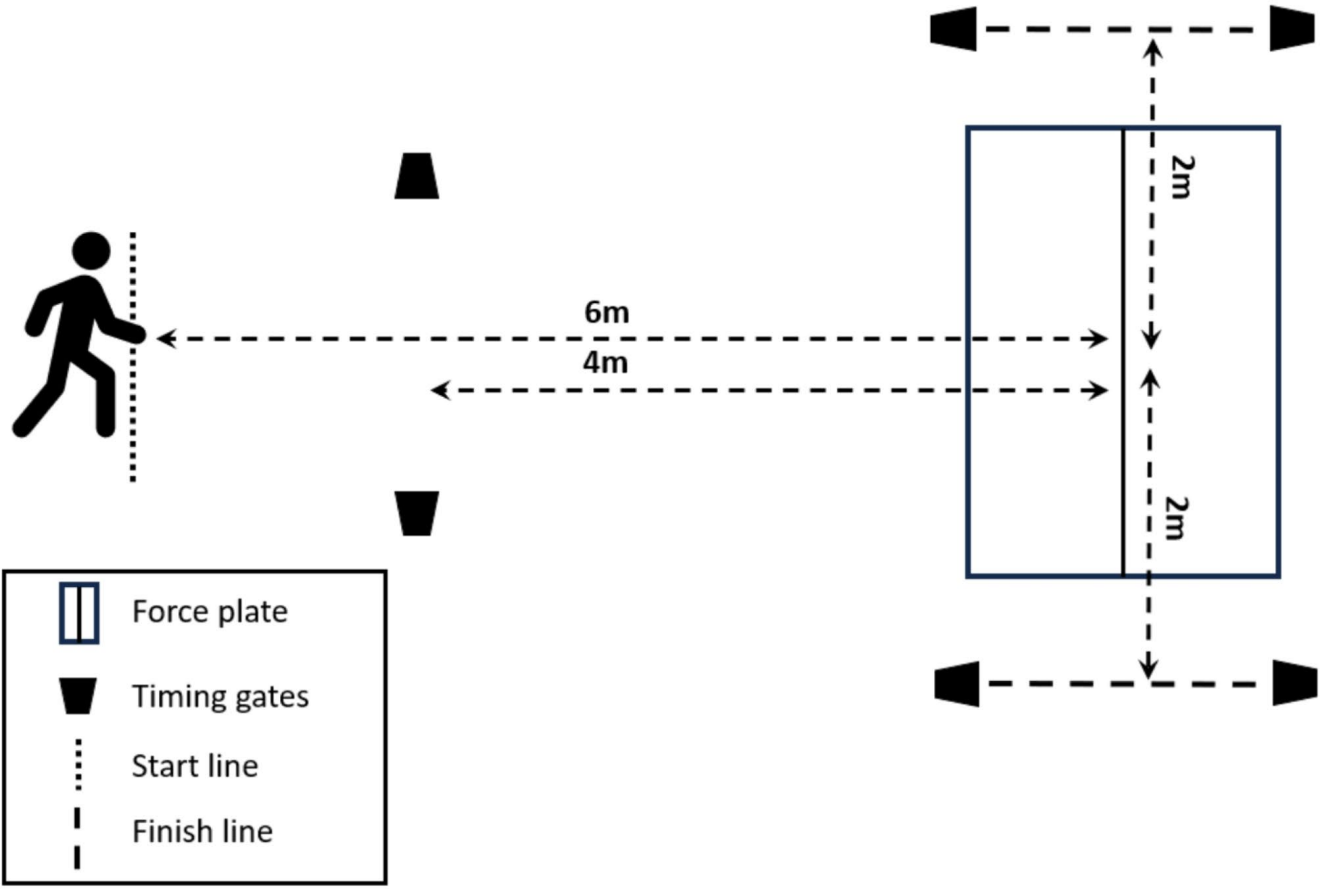
Laboratory setup for the 90°COD.

To evaluate the 90°COD, photoelectric sensors were positioned four meters in front of the force plate’s center, with an additional set-up two meters to each side (Fig. 2). Participants initiated their movement six meters ahead of the force plate’s center, thus enabling a 2 m approach before the first set of sensors. To perform a valid COD, the last foot contact had to be made within a specified 70 × 100 cm zone on the force plate. This contact was to be established with the right foot for the leftward change of direction, followed by an immediate 90° pivot to the left, and a quick progression through the second set of photoelectric sensors positioned two meters away. Conversely, for a rightward change of direction, contact was made using the left foot. Some flexibility was allowed in the exact starting point to accommodate the different stride patterns of each participant as they approached the force platform. Each participant was instructed to move toward the force platform on a straight path, to avoid making a premature turn. For the analysis, only trials that matched these conditions were included. Participants performed four valid trials on each side.

#### Multidirectional sprint performance (MS)

**Fig. 3:**
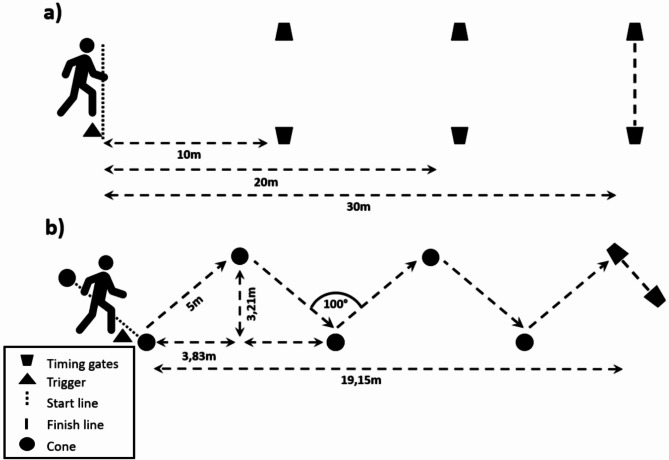
Setup of the multidirectional (**a**) and linear (**b**) sprint.

For the evaluation of the multidirectional sprinting ability, a standardized course was set up. One cone was placed every 3.83 m along a previously marked straight line on the field. Subsequently, every second cone was moved upward by 3.21 m, resulting in an approximate angle of 100° between each 5 m section from cone to cone (Fig. 3a). This arrangement of cones facilitated the required change of direction angle within the course. In total, the test covers a sprint distance of 25 m with four cutting maneuvers.

The TCi Motion Start was placed on their front leg, which triggered the timing when their foot was released from the ground. A pair of light barriers were placed at the end of the course to stop the final time. Participants started the test independently from a high start and were instructed to run through the course and around the cones as fast as possible. All participants completed three valid trials.

#### Linear sprint performance (LS)

To analyze the LS performance, two 30 m linear sprints with high start were conducted (Fig. 3b). Light barriers were placed at every 10 m interval. The TCi Motion Start was placed on their front leg, which triggered the timing when their foot was released from the ground. The participants were instructed to run as fast as possible and completed three valid trials.

### Data analysis

The jump data from the force plates were captured and exported to a text file with the Qualisys Track Manager (Version 2019.2, QTM, Gothenburg, Sweden). Force data and time data processing were done in Matlab (Mathworks, USA). The force data were filtered using a 4th order, zero-lag Butterworth low pass filter with a cutoff frequency of 50 Hz^50^. The signals from the two force plates were then summed. The resulting force curve was used to determine the timing of the takeoff and landing, thus allowing the calculation of the flight time. The times were determined by exceeding or falling below a force threshold of 70 N. This threshold was selected to facilitate the automated data analysis process and include jump trials with minor noise at the beginning of the movement. The jump height was calculated based on the flight time using the following formula^51^:

$$\:Jump\:height=g*{t}_{flight}^{2}*{8}^{-1}$$

**Fig. 4:**
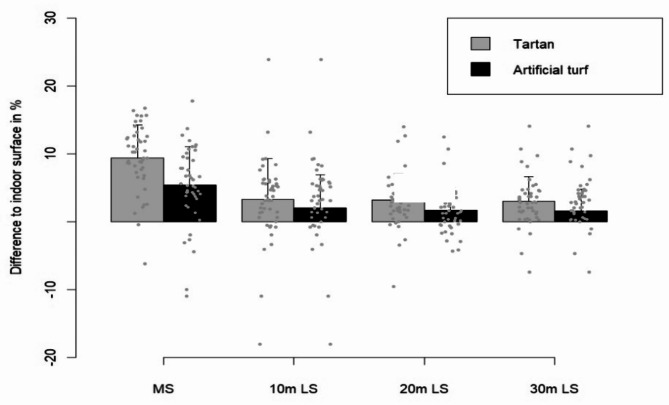
Mean percentage performance difference of LS and MS on artificial turf and tartan in relation to indoor surface.

Thereby *g* represents the acceleration due to gravity (9.81 m s^− 1^) and $\:{t}_{flight}$ the flight time. The mean jump height was computed from three trials of jumps for each footwear condition. Similarly, the average time values for the 90°COD were derived from the best three trials. For the LS and MS, the average times were calculated based on the two best trials (Fig. 4).

#### Statistics

Statistical analysis of the results was conducted using RStudio software, version 4.0.3 (RStudio, Boston). A two-factorial ANOVA with repeated measures was executed using the “aov_ez” command from the “afex” package^52^. Due to anticipated sex differences in the within-subject variables, sex was incorporated as a between-subject variable in the analyses. Additionally, the packages “lme4”^53^ and “Matrix”^54^ were loaded as prerequisites for computations and models during the analysis. The within-subject variables included the footwear and surface conditions. These variables varied according to the specific test. The Mauchly test was employed to check for sphericity, with Greenhouse-Geisser corrections applied when sphericity assumptions were not met. The effect sizes were calculated using eta-squared (η^2^): 0.01 for small effects, 0.06 for medium effects, and 0.14 for large effects^55^. For significant effects, Bonferroni post-hoc tests were carried out. A p-value < 0.05 was considered statistically significant. The percentage difference between the shoe and surface conditions was calculated using the following formula:

$$\:Percentage\:difference=\:\frac{Condition\:A-Condition\:B}{Condition\:A}$$

## Results

Forty-nine participants were initially enrolled (Table 1). Due to injuries or unspecified reasons, four participants were unable to complete at least one of the tests. Specifically, one female participant was missing the laboratory and indoor session and was therefore excluded from all analyses. In addition, two female and male participants did not complete the tartan track test session. As a result, these participants were excluded from the specific analyses.

### 90° change of direction performance

**Fig. 5:**
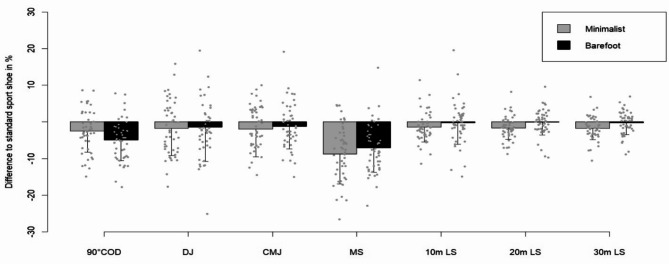
Mean percentage performance difference of all tests with minimalist shoe and barefoot condition in relation to the standard sport shoes.

A total of 46 participants were included in the footwear analysis. Footwear significantly influenced 90°COD (*F*(2,88) = 12.026, *p* < 0.001, *η*^2^ = 0.21). Post-hoc tests revealed that participants were faster while wearing the standard sport shoes compared to minimalist shoes (2.52%, *p* = 0.02) and in the barefoot condition (4.98%, *p* < 0.001) (Fig. 5). In addition, the minimalist shoe condition resulted in significantly faster COD times than the barefoot condition (2.59%, *p* = 0.045).

Sex did not significantly impact the 90°COD (*F*^1,44^ = 0.582, *p* = 0.45, *η*^2^ = 0.01).

### Jump performance

**Fig. 6:**
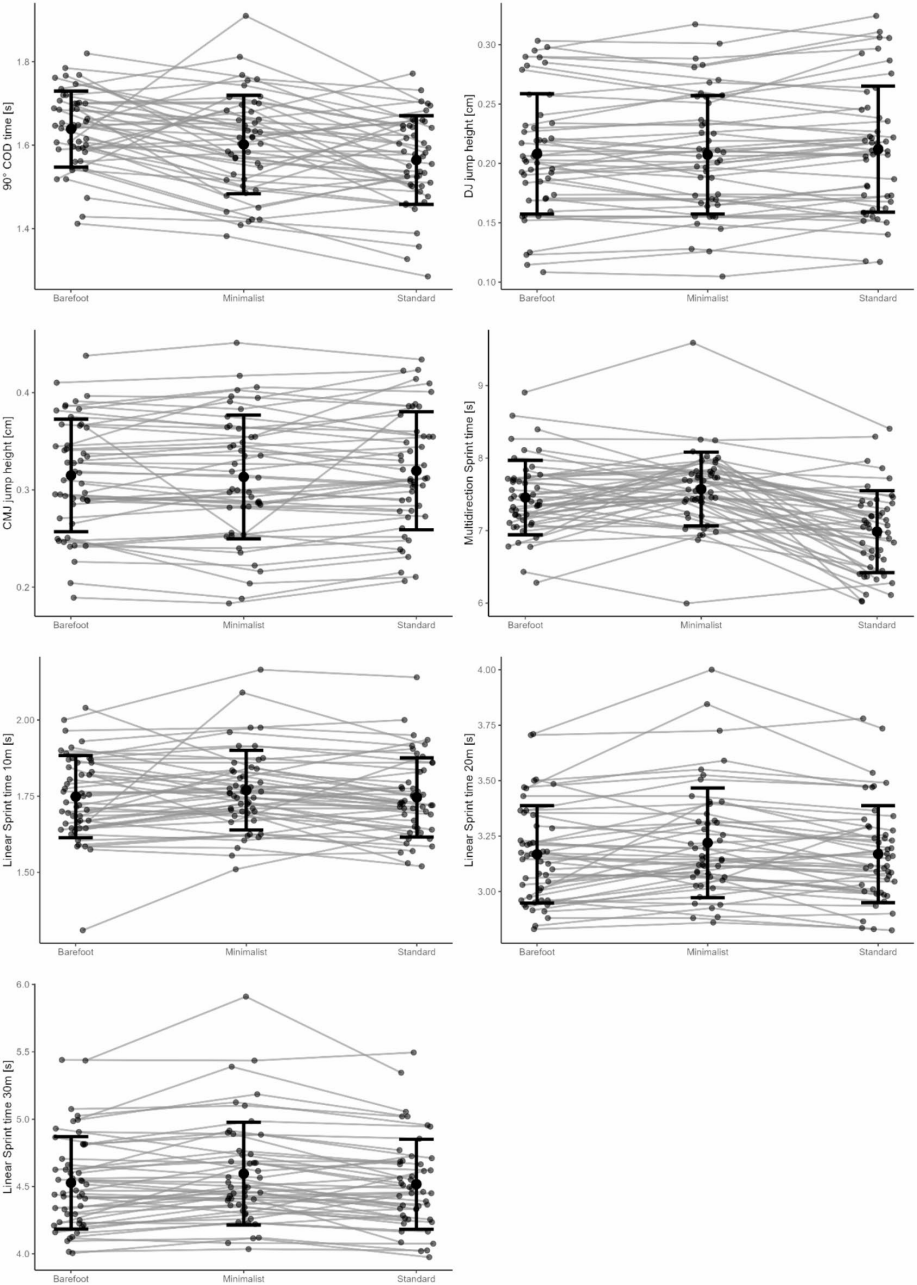
Comparison of mean and standard deviation of change of direction, jump heights, and sprint times across different footwear conditions (barefoot, minimalist, standard).

A total of 47 participants were included in the footwear analysis. No significant footwear effects were observed in the DJ height (*F*(2,90) = 2.805, *p* = 0.066, *η*^2^ = 0.06) or the CMJ height (*F*(2,90) = 1.666, *p* = 0.199, *η*^2^ = 0.04) (Fig. 6). A significant gender difference was observed in the DJ (*F*^1,45^ = 11.33, *p* = 0.002, *η*^2^ = 0.20) and CMJ (*F*^1,45^ = 15.247, *p* < 0.001, *η*^2^ = 0.25) (Table 2).

**Table 2:** Mean ± standard deviation of performance metrics across sex, footwear conditions and p-values of the ANOVA and post-hoc test.

		Women	Male	All	ANOVA *p* - value
90°COD (s)					
Footwear	Standard	1.59 ± 0.11	1.55 ± 0.11	1.56 ± 0.11	*p* < 0.001^b^
	Minimalist	1.62 ± 0.13	1.59 ± 0.11	1.60 ± 0.11	
	Barefoot	1.64 ± 0.08	1.64 ± 0.10	1.64 ± 0.09	
DJ^d^ (m)					
Footwear	Standard	0.18 ± 0.05	0.23 ± 0.05	0.21 ± 0.05	*p* = 0.066
	Minimalist	0.18 ± 0.05	0.23 ± 0.04	0.21 ± 0.05	
	Barefoot	0.18 ± 0.05	0.22 ± 0.04	0.21 ± 0.05	
CMJ^d^ (m)					
Footwear	Standard	0.28 ± 0.06	0.34 ± 0.05	0.32 ± 0.06	*p* = 0.199^GG^
	Minimalist	0.27 ± 0.07	0.34 ± 0.05	0.31 ± 0.06	
	Barefoot	0.28 ± 0.06	0.34 ± 0.05	0.32 ± 0.06	
MS^d, e^ (s)					
Footwear	Standard	7.43 ± 0.44	6.74 ± 0.48	6.99 ± 0.57	*p* < 0.001^a, b^
	Minimalist	7.78 ± 0.58	7.46 ± 0.44	7.57 ± 0.51	
	Barefoot	7.78 ± 0.53	7.28 ± 0.42	7.46 ± 0.51	
10 m LS^d^ (s)					
Footwear	Standard	1.83 ± 0.14	1.70 ± 0.10	1.75 ± 0.13	*p* = 0.266^GG^
	Minimalist	1.84 ± 0.14	1.73 ± 0.11	1.77 ± 0.13	
	Barefoot	1.84 ± 0.13	1.70 ± 0.11	1.75 ± 0.14	
20 m LS^d^ (s)					
Footwear	Standard	3.33 ± 0.24	3.08 ± 0.15	3.17 ± 0.22	*p* = 0.002^a, c^
	Minimalist	3.39 ± 0.30	3.12 ± 0.15	3.22 ± 0.25	
	Barefoot	3.33 ± 0.23	3.08 ± 0.15	3.17 ± 0.22	
30 m LS^d^ (s)					
Footwear	Standard	4.77 ± 0.36	4.38 ± 0.23	4.52 ± 0.33	*p* = 0.001^a, c^
	Minimalist	4.88 ± 0.45	4.44 ± 0.22	4.60 ± 0.38	
	Barefoot	4.79 ± 0.36	4.38 ± 0.23	4.53 ± 0.34	

^a^Post hoc test significantly different (*p* < 0.05) between standard sport and minimalist shoes.

^b^Post hoc test significantly different (*p* < 0.05) between standard sport shoes and barefoot.

^c^Post hoc test significantly different (*p* < 0.05) between minimalist shoes and barefoot.

^d^ANOVA significantly different (*p* < 0.05) between sex.

^e^ANOVA significant interaction effect (*p* < 0.05) between sex and footwear.

^GG^ Greenhouse-Geisser corrections applied to correct for sphericity.

*90°COD* 90° change of direction, *CMJ* countermovement jump, *DJ* single leg drop jump, *MS* multidirectional sprint, *LS* linear sprint.

### Multidirectional sprint performance

A total of 48 participants were included in the footwear analysis. The results demonstrated that footwear significantly influenced MS (F(2,92) = 34.474, *p* < 0.001, η^2^ = 0.43). Post-hoc tests indicated that wearing the standard sport shoes resulted in significantly faster times compared to the minimalist shoe (8.79%, *p* < 0.001) and barefoot condition (7.04%, *p* < 0.001). No difference was observed between the minimalist shoe and the barefoot sprinting (*p* = 0.18) (Table 2).

**Fig. 7:**
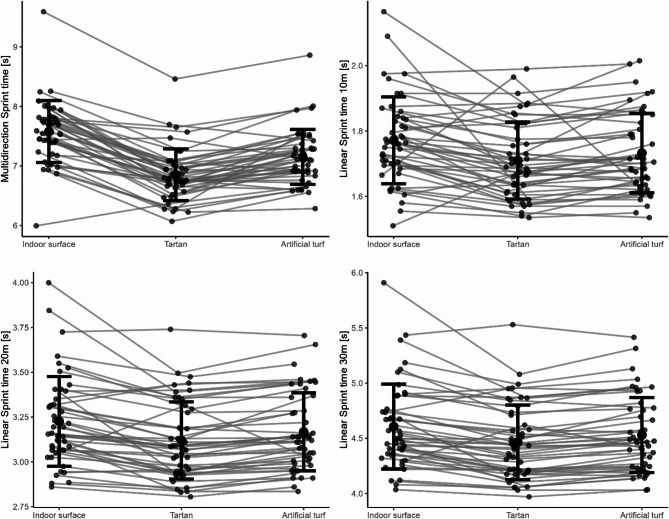
Comparison of mean and standard deviation of multidirectional and linear sprint times over 10 m, 20 m, and 30 m on different surfaces (indoor, tartan, artificial turf).

A total of 46 participants were included in the surface analysis. The surface conditions were found to significantly influence MS (F(2,88) = 79.22, *p* < 0.001, η^2^ = 0.64) (Fig. 7). Post-hoc tests showed that participants were faster on tartan by approximately 9.41% (*p* < 0.001) and on artificial turf by about 5.42% (*p* < 0.001) when compared to the indoor condition (Fig. 4). In addition, faster times were found on the tartan compared to the artificial turf (4.10%, *p* < 0.001) (Table 3).

**Table 3:** Mean ± standard deviation of performance metrics across sex, surface conditions and p-values of the ANOVA and post-hoc test.

		Women	Male	All	ANOVA *p*-value
MS^d^ (s)					
Surface	Indoor	7.78 ± 0.58	7.46 ± 0.46	7.58 ± 0.52	*p* < 0.001^a, b,c, GG^
	Artificial turf	7.47 ± 0.49	6.97 ± 0.33	7.15 ± 0.46	
	Tartan	7.15 ± 0.47	6.68 ± 0.31	6.85 ± 0.43	
10 m LS^d^ (s)					
Surface	Indoor	1.84 ± 0.14	1.73 ± 0.11	1.77 ± 0.13	*p* < 0.001^a, b,GG^
	Artificial turf	1.82 ± 0.13	1.68 ± 0.09	1.73 ± 0.12	
	Tartan	1.79 ± 0.11	1.67 ± 0.10	1.71 ± 0.12	
20 m LS^d^ (s)					
Surface	Indoor	3.39 ± 0.30	3.13 ± 0.16	3.23 ± 0.25	*p* < 0.001^a, b,c, GG^
	Artificial turf	3.32 ± 0.22	3.08 ± 0.16	3.17 ± 0.22	
	Tartan	3.27 ± 0.22	3.03 ± 0.16	3.12 ± 0.22	
30 m LS^d^ (s)					
Surface	Indoor	4.88 ± 0.45	4.45 ± 0.23	4.61 ± 0.39	*p* < 0.001^a, b,c, GG^
	Artificial turf	4.77 ± 0.35	4.39 ± 0.25	4.53 ± 0.34	
	Tartan	4.71 ± 0.36	4.32 ± 0.23	4.46 ± 0.34	

^a^Post hoc test significantly different (*p* < 0.05) between indoor and artificial turf.

^b^Post hoc test significantly different (*p* < 0.05) between indoor and tartan.

^c^Post hoc test significantly different (*p* < 0.05) between artificial turf and tartan.

^d^ANOVA significantly different (*p* < 0.05) between sex.

^GG^ Greenhouse-Geisser corrections applied to correct for sphericity.

*MS* multidirectional sprint, *LS* linear sprint.

There was a significant interaction effect between sex and footwear condition for MS performance (*F*(2.92) = 3.654, *p* = 0.03, *η*^2^ = 0.07).

### Linear sprint performance

A total of 48 participants were included in the footwear analysis. For the first 10 m of the 30 m LS, no significant effect on the footwear was observed (*F*(2,92) = 1.34, *p* = 0.266, *η*^2^ = 0.03) (Table 2). A total of 46 participants were included in the surface analysis. Surface conditions significantly impacted the first 10 m LS (*F*(2.88) = 8.844, *p* < 0.001, *η*^2^ = 0.17). Post-hoc tests revealed that the first 10 m LS were 3.27% faster on tartan (*p* = 0.0016), while artificial turf showed a 2.05% improvement (*p* = 0.021) compared to the indoor surface. No significant difference was observed between tartan and artificial turf (*p* = 0.105) (Table 3).

For the first 20 m of the 30 m LS, footwear had a significant effect on sprinting times (*F*(2.92) = 6.755, *p* = 0.002, *η*^2^ = 0.13). Post-hoc analyses exposed that wearing minimalist shoes resulted in longer times compared to the standard sport shoe (1.60%, *p* = 0.004) and the barefoot condition (1.40%, *p* = 0.013). No statistical difference was detected between the barefoot condition and standard sport shoe (*p* = 1.00) (Table 2). Surface conditions exhibited a significant influence on the first 20 m LS (*F*(2.88) = 20.175, *p* < 0.001, *η*^2^ = 0.31). Post-hoc tests showed that, compared to the indoor condition, sprinting on tartan led to an improvement of approximately 3.17% (*p* < 0.001) and on artificial turf of about 1.70% (*p* = 0.004). In addition, LS on tartan was superior to artificial turf (1.49%, *p* < 0.001) (Table 3).

The end of the 30 m LS was also significantly affected by footwear (*F*(2.92) = 8.678, *p* < 0.001, *η*^2^ = 0.16). Post-hoc tests revealed that participants were 1.72% faster with standard sport shoes than with minimalist shoes (*p* = 0.002). Additionally, the barefoot condition resulted in 1.31% faster times than the minimalist shoe condition (*p* = 0.008). However, there was no significant LS difference between barefoot condition and standard sport shoes (*p* = 1.00) (Table 2)The surface conditions significantly impacted the end of the 30 m LS (*F*(2.88) = 20.595, *p* < 0.001, *η*^2^ = 0.32). Post-hoc tests indicated that sprinting on tartan resulted in an approximate improvement of 2.97% (*p* < 0.001) and 1.56% on artificial turf (*p* = 0.007) in comparison to the indoor condition. Also sprinting on tartan showed an improvement compared to artificial turf (1.43%, *p* < 0.001) (Table 3).

## Discussion and implications

The aim of this study was to investigate the acute effects of minimalist footwear on performance during athletic movements as well as its interaction with sports-related surfaces. The study conducted tests on a range of athletic movements that are applicable to most sports. The results of the study aligned with our main hypothesis, revealing a significant acute effect of both footwear and surface conditions on performance.

### Footwear effect on change of direction performance

The study showed a reduction in performance in minimalist footwear and barefoot condition for 90°COD and MS compared to standard shoes. The loss of performance attributed to the footwear during a single 90° cutting maneuver was between 2 and 5%, whereas the MS, consisting of four 100° cutting maneuvers, showed a greater loss of performance of approximately 8%, which could be attributed to the multiple performance of the cutting maneuver, increasing the effect. Multidirectional movements involve higher forces than running, especially in a horizontal direction^56^. Modern athletic footwear is designed to support such movements^10^, while minimalist footwear aims to minimize interference with the natural movement of the foot through a thin, flexible sole and upper configuration^6^. Studies have shown that acute change from standard sports to minimalist footwear and barefoot running can affect various kinetic and kinematic parameters, including impact parameters^7^ and the foot strike angle^57^. It is accepted that a shock-absorbing and cautious movement pattern is acutely adapted by changing to minimalist footwear or barefoot conditions in order to reduce the impact forces^57–59^. Changes in ankle kinematics and impact parameters were also reported in cutting maneuvers when transitioning to minimalist footwear or a barefoot condition. Sinclair^35^ conducted a comparison of biomechanics between minimalist, court, energy return, and conventional sports footwear in nine participants during a 180° cutting maneuver. They observed significant changes in ankle kinematics in all three planes when wearing minimalist shoes. Consistent with the previous study of this research group on 45° cutting maneuver^29^, the foot strike was executed with a more plantarflexed ankle joint, resulting in a greater total ankle joint excursion. Despite this kinematic adaptation, the authors measured higher impact parameters in minimalist shoes. Similarly, Bisesti, Lawrence^36^ found that athletes performing the 45° cutting maneuver barefoot had a more anterior foot strike than with standard sports footwear. One difference between the minimalist and standard sport shoes used in our study is the cushioning properties. The weaker cushioning properties of the minimalist shoes may have led to an acute adaptation of movement patterns^60^. This adaptation could have been reflected in a more cautious and slower execution of the cutting maneuver to reduce the impact forces thus resulting in a loss of performance in both 90°COD and MS. In contrast to our study, referenced studies^29,35,36^ prescribed a standardized speed for cutting maneuvers to ensure biomechanical comparability. This constraint may have prevented participants from adapting their movement patterns, potentially resulting in higher impact loads. Moreover, an interaction effect between sex and shoe condition for MS on indoor surfaces, partially confirms our hypothesis of a sex-specific response. Women showed no difference between the barefoot condition and minimalist footwear. One possible explanation might be that women have less strength capacity, which requires less traction between the shoe and the surface. However, it is also possible that women were more cautious in their overall response to the unfamiliar situation.

### Footwear effect on sprint performance

The results of the study showed significant differences in the first 20 m and the end of the 30 m LS performances between sprinting barefoot and with standard sport shoes compared to minimalist shoes. However, no significant differences in performance were observed between the different footwear conditions for the first 10 m sprint distances. The study found that the use of minimalist shoes resulted in a performance loss of approximately 1%. Only two studies have investigated the effect of barefoot sprinting compared to spiked shoes, with biomechanical parameters as the primary outcome rather than performance. Smith, Lake^32^ reported a 4% increase in sprint speed over a 20 m distance when wearing spikes. Toon, Williams^31^ did not provide specific performance data. Both studies found a stiffer metatarsophalangeal joint at the end of the stance phase, which may explain the performance improvement. However, we could not find any difference between a standard sports shoe and the barefoot condition, indicating this explanation does not apply to our results. In contrast to the studies considered, which investigated sprinters wearing spikes, our study focused on recreational athletes and standard sports shoes. Our findings suggest that the negative effect becomes measurable only after several steps and at higher speeds. Since the participants did not react negatively to the unfamiliar barefoot condition, the specific material properties of the minimalist shoe might be responsible.

### Footwear effect on jump performance

Our study did not find a significant effect of footwear on CMJ nor on DJ performance, which is consistent with the results of existing studies^24,25,61^. Conversely, LaPorta, Brown^27^ reported a significant improvement in CMJ performance when using minimalist footwear or when jumping barefoot, compared to wearing tennis shoes. Also, Sinclair, Toth^28^ found significant differences in jump height between the shoe conditions, with the minimalist and conventional shoe conditions performing worse than the energy return shoes in a 40 cm depth jump with a 30 cm horizontal offset using ten participants. The minimalist shoes showed lower impact parameters without a change in ankle kinematics, which can be attributed to the lower jump height. Possible reasons for the divergent results compared to the present study could be the different footwear used, the study population, and the execution of the drop jump. However, Harry, Eggleston^24^ conducted a single-subject analysis on 15 participants to evaluate the impact of footwear on CMJ using force plates and electromyography. The authors found a highly individualized response to footwear changes regarding muscle activity and performance. While our study uses a group-level analysis, these findings underscore the potential variability in individual responses, emphasizing the need for further research to explore such differences.

### Surface effect on performance

Participants exhibited better performance in minimalist footwear compared to the barefoot condition during the 90°COD. In contrast, superior performance was observed in the barefoot condition for the MS on the indoor surface, suggesting a possible surface interaction effect that may favor the barefoot condition on the indoor surface. Moreover, the MS and LS demonstrated significant performance improvements in the change from indoor surfaces to tartan and artificial turf when using minimalist footwear. In particular, the LS showed an average increase in performance of approximately 3%, while the MS exhibited an improvement of approximately 8% compared to indoor surfaces. The results suggest that not only the unfamiliarity but also the properties of the minimalist footwear are responsible for the performance effects. These properties interact better with artificial turf and tartan track than with the indoor surface, leading to improved performance. Worobets and Wannop^18^ concluded that outsole traction had the largest influence on sprinting, jumping with a running approach, and change of direction performance compared to forefoot bending stiffness and shoe weight, as the participants performed significantly worse in all tests when traction was decreased by 20%. Several studies have supported the importance of traction in athletic performance, especially in change of direction movements^9,14,18^. The mechanism behind the performance improvement from traction may be that it allows the athlete to lean more into the surface and direct the GRF more effectively in the desired direction, resulting in a potentially higher horizontal GRF^14^. The orientation of the GRF correlates closely with acceleration performance^12^. However, other surface and shoe properties could also have an impact on performance, such as surface compliance^9^, shoe upper configuration^19^, and inner sole traction^20^. Therefore, the improvements in performance parameters can be attributed to the interaction between specific material properties of the surfaces and the shoe. However, it should be noted that the observed performance improvements with minimalist footwear on different surfaces may not necessarily apply to standard footwear or barefoot conditions, as these were not tested across different surfaces in this study.

### Limitations

There are some limitations to this study. The different tests were conducted on separate days and under varying environmental conditions (indoor and outdoor), which limits the comparability between measurements, at least for the surface comparison. However, since each test with all shoe conditions was completed on the same day, we did not expect any influence on the footwear effect. Another limitation of the study is the lack of direct measurements of the material properties of the footwear and surfaces. As a result, only speculation can be made as to the underlying cause of the differences in performance between the shoes and surfaces. Furthermore, the results of this study are specific to the particular minimalist shoes tested, which limits the generalizability of our findings to other models or brands. Moreover, only the minimalist footwear condition was examined across different surface conditions. This restricts our ability to fully compare the effects of standard footwear and barefoot conditions on various surfaces and limits the generalizability of our findings regarding surface interactions. Future studies should include all footwear conditions across different surfaces to comprehensively assess these interactions. Additionally, it should be noted that the findings of this study are restricted to individuals who habitually wear shoes. Populations already accustomed to running in minimalist footwear or barefoot, or athletes from barefoot sports (e.g. martial arts, gymnastics) may respond differently to the footwear transition^58^. Finally, further studies should investigate the long-term effects of minimalist footwear on performance, as the acute effects of changing to minimalist footwear may be a response to an unfamiliar condition and cannot be directly generalized to long-term adaptations^7^. Finally, testing conditions, including different types of sport and athletic demands, should be considered in future studies to improve the applicability of findings across a wider range of athletic populations. Investigating the interplay between footwear, surfaces, and sport-specific movements could provide further insights into optimizing athletic performance and injury prevention.

### Practical implications

The results of the study have implications for the selection of competitive footwear, performance assessment and training of athletes, as well as for manufacturers of minimalist footwear. Particularly in disciplines that require fast COD, minimalist and barefoot conditions are not the optimal footwear choices. An abrupt change to these conditions should therefore be avoided for performance purposes. However, the use of minimalist footwear for linear movements and jumps can be recommended. The results showed that footwear and surface condition have an influence on test results and should therefore be considered in performance assessments. Despite the acute performance loss when wearing minimalist shoes or barefoot, there is evidence suggesting potential positive long-term effects on both performance^62–64^ and health factors^3,6^. To make minimalist footwear more attractive to athletes, manufacturers should consider modifying the sole and upper properties. This is a difficult challenge as it could restrict the natural movement of the foot.

## Conclusion

Minimalist footwear and barefoot conditions showed a significant acute negative effect on performance in MS, LS, and 90°COD under the tested conditions. There was no effect on jumping performance. Depending on the surface and movement, performance losses of up to 9% were observed in minimalist compared to standard sport footwear. However, on tartan, minimalist shoes achieved comparable performance results in MS and LS to standard sport footwear on indoor surfaces, likely due to the material properties of the shoe and surface providing better traction. Additionally, there was an indication of sex-specific responses to the shoe change in the MS. These findings suggest that the effects of minimalist footwear are context-dependent and should not be generalized without considering specific surfaces, movements, and individual factors.

## Electronic supplementary material

Below is the link to the electronic supplementary material.

Supplementary Material 1

## Data Availability

The datasets used and/or analysed during the current study are available from the corresponding author on reasonable request.
