# Supplementary material for: Acute effects of footwear and surface condition on sport specific performance in athletes
Source: Sci Rep. 2025 Feb 26;15:6969. doi: 10.1038/s41598-025-91515-w (PMC11865527; doi:10.1038/s41598-025-91515-w)
Supplement: Supplementary file 1 — Supplementary Material 1 [file 41598_2025_91515_MOESM1_ESM.docx]

# Appendix

Tab. S1: Shoe type, brand and model of each subject standard sport shoes

| **Participant** | **Shoe type** | **Brand** | **Modell** |
| --- | --- | --- | --- |
| 1 | Indoor shoe | Adidas | Sala |
| 2 | Casual shoe | Lico | Limber |
| 3 | Indoor shoe | Nike | Jordan Westbrook One Take |
| 4 | Running shoe | Evadict | Trail XT7 |
| 5 | Running shoe | Asics | GEL-Fuji Attack 2 |
| 6 | Running shoe | Adidas | Ultraboost 20 |
| 7 | Indoor shoe | Yonex | Badminton shoe |
| 8 | Running shoe | New Balance | Fresh Foam |
| 9 | Running shoe | Asics | Gel Cumulus 17 |
| 10 | Indoor shoe | Adidas | Hb Special Pro |
| 11 | Running shoe | Adidas | NMD |
| 12 | Running shoe | Hoka | One One Torrent |
| 13 | Running shoe | Adidas | Alphabounce |
| 14 | Running shoe | Puma | NRGY Star Multiknit |
| 15 | Running shoe | Brooks | Ravenna |
| 16 | Running shoe | New Balance | Fresh Foam |
| 17 | Running shoe | Nike | Flex Trainer |
| 18 | Running shoe | Nike | Revolution 5 |
| 19 | Running shoe | Brooks | Ghost 13 |
| 20 | Running shoe | Nike | Legend react 2 AT |
| 21 | Running shoe | Adidas | Ultraboost |
| 22 | Running shoe | Adidas | Ultraboost |
| 23 | Running shoe | Nike | Renew ride |
| 24 | Running shoe | Nike | Air Zoom Vomero |
| 25 | Running shoe | Adidas | Ultraboost |
| 26 | Running shoe | Nike | Air Zoom Terra Kiger |
| 27 | Running shoe | Nike | Legend react |
| 28 | Running shoe | Adidas | Adizero Adios 5 |
| 29 | Running shoe | Brooks | Glycerin |
| 30 | Indoor shoe | Nike | Tiempo Mystic IV |
| 31 | Running shoe | Nike | Joyride run flyknit |
| 32 | Running shoe | Adidas | Ultraboost |
| 33 | Running shoe | Nike | Legend react |
| 34 | Casual shoe | Nike | Air Force |
| 35 | Running shoe | Adidas | Ultraboost |
| 36 | Running shoe | Nike | Flex Trainer |
| 37 | Running shoe | Yonex | Power Cushion |
| 38 | Running shoe | Nike | Lunargato |
| 39 | Running shoe | Asics | Gel-Pulse |
| 40 | Running shoe | Nike | Air Zoom Pegasus |
| 41 | Running shoe | ON | Running Cloud |
| 42 | Running shoe | Nike | Legend react |
| 43 | Running shoe | Nike | epic react flyknit |
| 44 | Running shoe | Adidas | Ultraboost |
| 45 | Running shoe | Nike | Performance Revolution |
| 46 | Running shoe | Nike | Zoom |
| 47 | Running shoe | Nike | Mercurial Vapor |
| 48 | Running shoe | Adidas | Ultraboost |
